# Baseline cardiovascular risk assessment in cancer patients scheduled to receive cardiotoxic cancer therapies: a position statement and new risk assessment tools from the Cardio-Oncology Study Group of the Heart Failure Association of the European Society of Cardiology in collaboration with the International Cardio-Oncology Society

**DOI:** 10.1002/ejhf.1920

**Published:** 2020-08-06

**Authors:** Alexander R. Lyon, Susan Dent, Susannah Stanway, Helena Earl, Christine Brezden-Masley, Alain Cohen-Solal, Carlo G. Tocchetti, Javid J. Moslehi, John D. Groarke, Jutta Bergler-Klein, Vincent Khoo, Li Ling Tan, Markus S. Anker, Stephan von Haehling, Christoph Maack, Radek Pudil, Ana Barac, Paaladinesh Thavendiranathan, Bonnie Ky, Tomas G. Neilan, Yury Belenkov, Stuart D. Rosen, Zaza Iakobishvili, Aaron L. Sverdlov, Ludhmila A. Hajjar, Ariane V.S. Macedo, Charlotte Manisty, Fortunato Ciardiello, Dimitrios Farmakis, Rudolf A. de Boer, Hadi Skouri, Thomas M. Suter, Daniela Cardinale, Ronald M. Witteles, Michael G. Fradley, Joerg Herrmann, Robert F. Cornell, Ashutosh Wechelaker, Michael J. Mauro, Dragana Milojkovic, Hugues de Lavallade, Frank Ruschitzka, Andrew J.S. Coats, Petar M. Seferovic, Ovidiu Chioncel, Thomas Thum, Johann Bauersachs, M. Sol Andres, David J. Wright, Teresa López-Fernández, Chris Plummer, Daniel Lenihan

**Affiliations:** 1 Cardio-Oncology Service, Royal Brompton Hospital and Imperial College, London, UK; 2 Duke Cancer Institute, Duke University, Durham, NC, USA; 3 Breast Unit, Royal Marsden Hospital, Surrey, UK; 4 Department of Oncology, University of Cambridge and NIHR Cambridge Biomedical Research Centre, Cambridge, UK; 5 Division of Medical Oncology, Sinai Health System, Mount Sinai Hospital, Toronto, Canada; 6 UMR-S 942, Paris University, Cardiology Department, Lariboisiere Hospital, AP-HP, Paris, France; 7 Department of Translational Medical Sciences and Interdepartmental Center for Clinical and Translational Research (CIRCET), Federico II University, Naples, Italy; 8 Cardio-Oncology Program, Department of Medicine, Vanderbilt University Medical Center, Nashville, TN, USA; 9 Cardio-Oncology Program, Brigham & Women’s Hospital, Harvard Medical School, Boston, MA, USA; 10 Department of Cardiology, Medical University of Vienna, Vienna, Austria; 11 Department of Clinical Oncology, Royal Marsden Hospital and Institute of Cancer Research, London, UK; 12 Department of Medical Imaging and Radiation Sciences, Monash University and Department of Medicine, Melbourne University, Melbourne, Australia; 13 Department of Cardiology, National University Heart Centre, Singapore, National University Health System, Singapore, Singapore; 14 Division of Cardiology and Metabolism, Department of Cardiology, Charité and Berlin Institute of Health Center for Regenerative Therapies (BCRT) and DZHK (German Centre for Cardiovascular Research), partner site Berlin and Department of Cardiology, Charité Campus Benjamin Franklin, Berlin, Germany; 15 Department of Cardiology and Pneumology, University of Goettingen Medical Center, Goettingen, Germany; 16 German Center for Cardiovascular Research (DZHK), partner site Goettingen, Goettingen, Germany; 17 Comprehensive Heart Failure Center, University Clinic Würzburg, Würzburg, Germany; 18 First Department of Medicine – Cardioangiology, Charles University Prague, Medical Faculty and University Hospital Hradec Kralove, Prague, Czech Republic; 19 MedStar Heart and Vascular Institute, Georgetown University, Washington, DC, USA; 20 Ted Rogers Program in Cardiotoxicity Prevention and Joint Division of Medical Imaging, Peter Munk Cardiac Center, Toronto General Hospital, University Health Network, University of Toronto, Toronto, Canada; 21 University of Pennsylvania, Philadelphia, PA, USA; 22 Cardio-Oncology Program, Division of Cardiology, Department of Medicine, Massachusetts General Hospital, Harvard Medical School, Boston, MA, USA; 23 Sechenov Medical University, Moscow, Russia; 24 Department of Community Cardiology, Tel Aviv Jaffa District, Clalit Health Fund and Sackler Faculty of Medicine, Tel Aviv University, Tel Aviv, Israel; 25 School of Medicine and Public Health, University of Newcastle and “Cancer and the Heart” Program, Hunter New England LHD, Newcastle, Australia; 26 Cardio-Oncology, Department of Cardio-Pneumology, University of São Paulo, São Paulo, Brazil; 27 Santa Cardio-Oncology, Santa Casa de São Paulo and Rede Dor São Luiz, São Paulo, Brazil; 28 Barts Heart Centre and University College London, London, UK; 29 Department of Precision Medicine, Luigi Vanvitelli University of Campania, Naples, Italy; 30 University of Cyprus Medical School, Nicosia, Cyprus; 31 Cardio-Oncology Clinic, Heart Failure Unit, “Attikon” University Hospital, National and Kapodistrian University of Athens Medical School, Athens, Greece; 32 Department of Cardiology, University of Groningen, University Medical Center Groningen, Groningen, The Netherlands; 33 Cardiology Division, Internal Medicine Department, American University of Beirut Medical Center, Beirut, Lebanon; 34 Department of Cardiology, Bern University Hospital, Inselspital, University of Bern, Bern, Switzerland; 35 Cardioncology Unit, European Institute of Oncology, IRCCS, Milan, Italy; 36 Stanford University School of Medicine, Stanford, CA, USA; 37 Department of Cardiovascular Diseases, Mayo Clinic, Rochester, MN, USA; 38 Vanderbilt University Medical Center, Nashville, TN, USA; 39 National Amyloidosis Centre, University College London, London, UK; 40 Memorial Sloan Kettering Cancer Center, New York, NY, USA; 41 Department of Haematology, Hammersmith Hospital, Imperial College, London, UK; 42 Department of Haematological Medicine, King’s College Hospital, London, UK; 43 University Heart Center, Department of Cardiology, University Hospital Zurich, Zurich, Switzerland; 44 University of Warwick, Warwick, UK; 45 Pharmacology, Centre of Clinical and Experimental Medicine, IRCCS San Raffaele Pisana, Rome, Italy; 46 Faculty of Medicine and Serbian Academy of Sciences and Arts, University of Belgrade, Belgrade, Serbia; 47 Emergency Institute for Cardiovascular Diseases ‘Prof. C.C. Iliescu’, Bucharest, Romania; 48 University of Medicine Carol Davila, Bucharest, Romania; 49 Institute of Molecular and Translational Therapeutic Strategies, Hannover Medical School, Hannover, Germany; 50 Department of Cardiology and Angiology, Hannover Medical School, Hannover, Germany; 51 Liverpool Centre for Cardiovascular Science, Liverpool Heart and Chest Hospital, Liverpool, UK; 52 Cardiology Service, Cardio-Oncology Unit, La Paz University Hospital and IdiPAz Research Institute, Ciber CV, Madrid, Spain; 53 Department of Cardiology, The Newcastle upon Tyne Hospitals NHS Foundation Trust and Newcastle University, Newcastle, UK; 54 Cardio-Oncology Center of Excellence, Washington University in St Louis, St Louis, MO, USA

**Keywords:** Risk factors, Cardio-oncology, Cardiotoxicity, Heart failure, Risk prediction

## Abstract

This position statement from the Heart Failure Association of the European Society of Cardiology Cardio-Oncology Study Group in collaboration with the International Cardio-Oncology Society presents practical, easy-to-use and evidence-based risk stratification tools for oncologists, haemato-oncologists and cardiologists to use in their clinical practice to risk stratify oncology patients prior to receiving cancer therapies known to cause heart failure or other serious cardiovascular toxicities. Baseline risk stratification proformas are presented for oncology patients prior to receiving the following cancer therapies: anthracycline chemotherapy, HER2-targeted therapies such as trastuzumab, vascular endothelial growth factor inhibitors, second and third generation multi-targeted kinase inhibitors for chronic myeloid leukaemia targeting BCR-ABL, multiple myeloma therapies (proteasome inhibitors and immunomodulatory drugs), RAF and MEK inhibitors or androgen deprivation therapies. Applying these risk stratification proformas will allow clinicians to stratify cancer patients into low, medium, high and very high risk of cardiovascular complications prior to starting treatment, with the aim of improving personalised approaches to minimise the risk of cardiovascular toxicity from cancer therapies.

## Introduction

There is a growing epidemic of cardiovascular disease (CVD) in cancer patients during and after cancer treatment. Improved cancer-related survival and the development of more targeted molecular therapies, in addition to the continued use of anthracycline chemotherapy, have resulted in a significant increase in both current and previously treated cancer patients presenting to cardiology services with CVD.^1^ The frequency of cardiovascular (CV) problems is higher in cancer patients who are receiving or who have previously received cancer treatments with a known CV toxicity profile. The average age of oncology patients is also increasing with the general ageing of the population, which is in part due to improved survival from CVD. Therefore, there are a rising number of patients who present to oncology and haemato-oncology services not only with a new cancer diagnosis but also with pre-existing CVD or risk factors for CVD.^1^ This poses a particular challenge when considering evidence-based oncology treatments that improve survival but impart a higher risk of CV toxicity.

**Figure 1:**
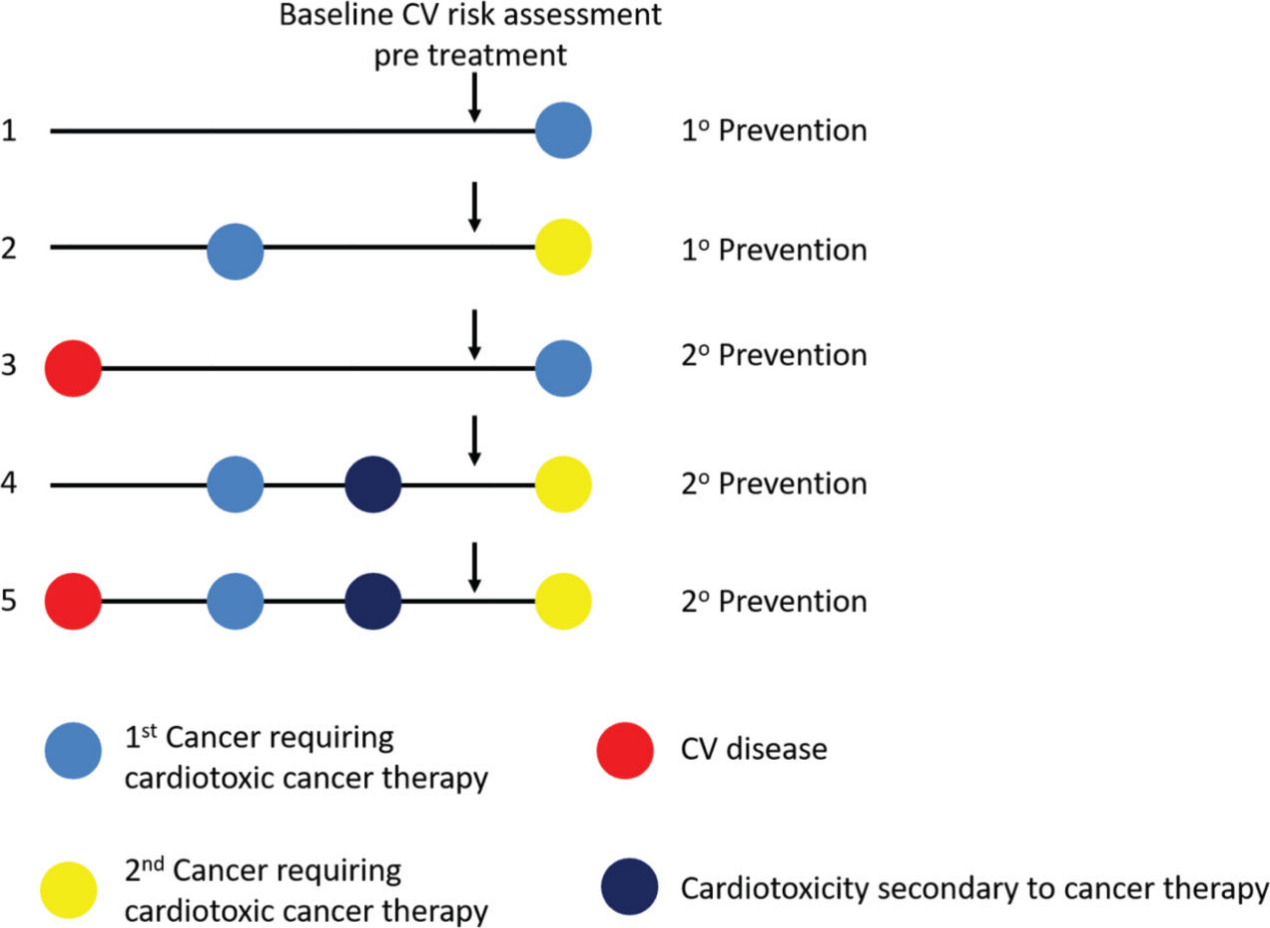
Examples of five different patients and primary or secondary prevention based on the history of pre-existing cardiovascular (CV) disease and/or prior cardiotoxicity from treatment of a previous malignancy.

Current oncology practice, including treatment planning and protocols for cancer treatments with potentially CV toxicity, provides unique opportunities to comprehensively assess CV health before initiation of cancer treatment. This allows cardiologists and other healthcare professionals, working in partnership with oncologists and haemato-oncologists, to optimise the management of pre-existing CVD and modifiable CV risk factors with the aim of reducing the risk of CV complications during and after cancer treatment. The assessment occurring prior to the initiation of cancer treatment and in patients without overt CVD or previous cardiotoxicity can be considered a *primary prevention* strategy while interventions in patients with pre-existing CVD or evidence of prior CV toxicity fall into the category of *secondary prevention* (Figure 1). Specialist care of CVD in cancer patients is now offered by dedicated cardio-oncology services which have emerged over the last 10 years.^2,3^ This multidisciplinary approach has the potential not only to reduce morbidity and mortality from CVD, but also to improve cancer outcomes by reducing interruptions in cancer treatment due to CV events and facilitate treatment options with greater potential CV risk. The aim of a multidisciplinary cardio-oncology approach is to support best practice, guideline-directed cancer care by maintaining cancer patients on effective therapies for as long as recommended, and increase the proportion of cancer patients who complete their cancer treatment without interruption for CVD.

**Figure 2:**
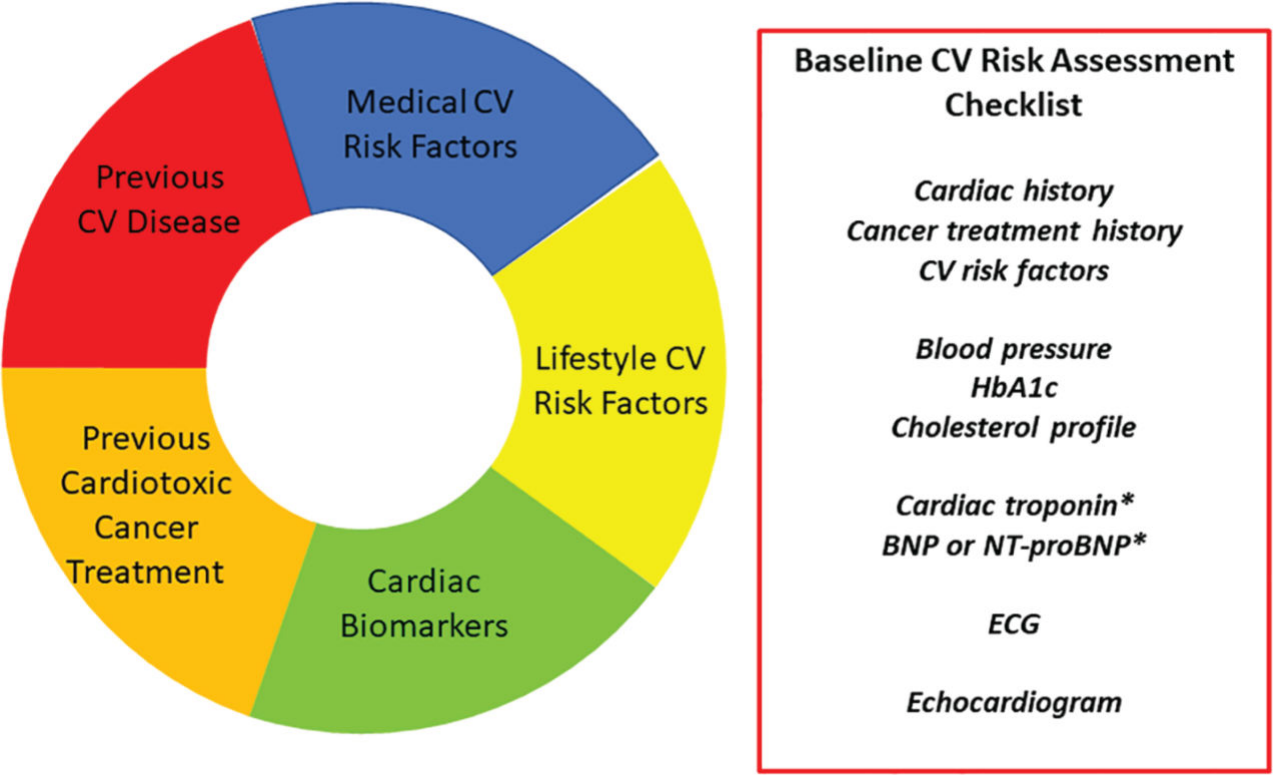
The different risk factors which contribute to baseline cardiovascular (CV) risk in a cancer patient scheduled to receive a cardiotoxic cancer treatment, and a checklist of the clinical history and investigations required at baseline prior to starting a cardiotoxic cancer therapy. *Cardiac biomarkers (troponin and natriuretic peptides) should be measured where available. BNP, brain natriuretic peptide; ECG, electrocardiogram; HbA1c, glycated haemoglobin; NT-proBNP, N-terminal pro-brain natriuretic peptide.

Many studies have identified a range of parameters that contribute to CVD risk, but these risk factors are not routinely and systematically assessed in oncology and haemato-oncology units at the time of cancer diagnosis or during cancer treatment (Figure 2). Several guidelines and expert position statements have been published by professional societies of cardiology, oncology and cardio-oncology focussed on CVD in cancer patients and all recommend baseline CV risk assessment in cancer patients prior to starting potentially cardiotoxic cancer treatments.^1,4–6^ However, there is no standardised system or risk assessment tool to facilitate CV risk stratification in oncology and haemato-oncology services.

The Cardio-Oncology Study Group from the Heart Failure Association (HFA) of the European Society of Cardiology (ESC) hosted a workshop in collaboration with the International Cardio-Oncology Society (ICOS) dedicated to the development of ‘Baseline CV risk stratification proformas’ that can be used by oncology and haemato-oncology teams to stratify cancer patients for CV risk before initiation of potentially cardiotoxic cancer therapies (Table 1). This position statement summarises the evidence reviewed at the workshop and subsequently by the co-authors of this paper, and proposes new HFA-ICOS baseline CV risk stratification proformas for seven classes of cancer therapies which are associated with significant risk of CVD, including but not limited to heart failure (HF).

## General principles

The assessment of baseline CV risk in cancer patients before starting potentially CV toxic cancer therapies is based on the following core principles:

- Risk is a continuous variable.
- Multiple CV risk factors may co-exist in an individual cancer patient and they have an additive or synergistic contribution to CV risk.
- Evidence or expert consensus exists that a parameter contributes to future risk of CVD and justifies its inclusion in the baseline CV risk assessment proforma.
- Increased absolute risk (rather than relative risk) is the most relevant for individual patient-based treatment decisions and the relative importance attributed to these risk factors.
- Cancer patients identified at increased risk of cancer treatment-related CV toxicity using these baseline CV risk assessment proformas should not have their evidence-based cancer treatment withheld unless they are identified at high or very high risk and after multidisciplinary discussion between the treating oncologist/haematologist and cardiologist.
- Baseline CV risk stratification should be completed promptly and should not delay starting cancer treatment (unless high or very high risk or pre-existing CVD is present).
- Decisions to withhold effective but potentially cardiotoxic cancer treatments in cancer patients at high or very high risk of CVD should only be made after multidisciplinary team discussion between the treating oncologist/haematologist and cardiologist balancing treatment efficacy vs. safety and CV risk for a particular individual.
- Decisions regarding switching to alternative less cardiotoxic cancer treatments in cancer patients identified at high or very high risk of CVD should only be made after multidisciplinary team discussion between the treating oncologist/haematologist and cardiologist, balancing treatment efficacy vs. safety and CV risk for a particular individual.
- The cancer patient should be informed and participate in the decision making process and be informed of their baseline CV risk level.
- Cardiovascular treatment interventions should be considered to mitigate CV risk in cancer patients when identified.
- Pathways of care should exist within an institution using these proformas so that patients with increased CV risk (medium, high or very high) have their pre-existing CVD and modifiable CV risk factors reviewed and optimised by a suitable healthcare professional (e.g. cardio-oncology service, cardiologist or primary care/family physician) depending on the nature of the risk, the cardiotoxic treatment planned and healthcare system.
- Baseline CV risk assessment proformas should be easy to understand and implement in oncology and haemato-oncology services.
- The application and impact of baseline CV risk assessment proformas can be assessed using an appropriate clinical audit and review.

## Design and application of baseline cardiovascular risk proformas

Several cancer drug therapies associated with clinically important rates of CV toxicity during or after treatment exposure are summarised in Table 1. The authors acknowledge that other cancer therapies are associated with CV risks (e.g. radiation therapy, stem cell transplantation); however, these are beyond the scope of this article. There is growing use of immune therapies in oncology, and there is now considerable evidence of CV toxicities from immune checkpoint inhibitors (ICIs) and emerging information of HF complicating cytokine release syndrome following chimeric antigen receptor T (CAR-T) cell therapies for various cancers.^7–9^ Whilst no evidence currently exists regarding which clinical, imaging and laboratory parameters may identify patients at higher risk, given the severity of these complications we recommend all patients scheduled to receive ICI or CAR-T therapy have a baseline echocardiogram, electrocardiogram (ECG) and measurement of cardiac troponin and a natriuretic peptide, which serve as a baseline reference if new cardiac complications develop.

Baseline CV risk assessment proformas have been developed for seven cardiotoxic cancer therapy classes known to cause a range of CV toxicities including left ventricular dysfunction (LVD) and HF (Tables 2–8).^10–68^ The risk is estimated for all CV complications from the drug class, e.g. left ventricular dysfunction, QTc prolongation and arrhythmias, vascular events including myocardial infarction (MI) and hypertension:

- *Anthracycline chemotherapy*: the main CV complications of anthracycline chemotherapy are LVD and HF, and atrial and ventricular arrhythmias.^19,69^
- *HER2 targeted therapies*: the main CV complications of HER2 targeted therapies are LVD and HF, and systemic hypertension.^44,70,71^
- *Vascular endothelial growth factor (VEGF) inhibitors [these agents are also known as angiogenesis inhibitors or VEGF tyrosine kinase inhibitors (TKIs) as many act via multi-targeted inhibition of tyrosine kinases]*: the main CV complications of VEGF inhibitors are systemic hypertension, LVD and HF, QTc prolongation and arterial thrombosis including MI.^48,53,57,72^
- *Multi-targeted kinase inhibitors for chronic myeloid leukaemia (CML) targeting BCR-ABL (often called BCR-ABL TKIs)*: the main CV complications of multi-targeted kinase inhibitors for CML targeting BCR-ABL include arterial thrombosis leading to MI, stroke and peripheral arterial occlusive disease (ponatinib), venous thromboembolism, systemic hypertension, LVD and HF, accelerated atherosclerosis (ponatinib and nilotinib), QTc prolongation (nilotinib) and pulmonary hypertension (dasatinib).^59,62,73–78^
- *Proteasome inhibitors (PIs) and immunomodulatory drugs (IMIDs)*: the main CV complications of PIs and IMIDs in combination are LVD and HF, ischaemia and MI, atrial and ventricular arrhythmias, venous thromboembolism and arterial thrombosis.^66,67,79^
- *Combination RAF and MEK inhibitor treatment*: the main CV complications of RAF and MEK inhibitors are LVD, HF and systemic hypertension for all combinations and QTc prolongation for one combination (vemurafenib and cobimetinib).^80,81^
- *Androgen deprivation therapies (ADT) for prostate cancer treatment including gonadotropin release hormone (GnRH) agonists*: ADT are associated with an increased risk of diabetes mellitus, hypertension and atherosclerosis (see below).^82–84^
- *Immune checkpoint inhibitors*: myocarditis including fulminant myocarditis, non-inflammatory HF, ventricular arrhythmias, atrio-ventricular block, sudden cardiac death, acute coronary syndromes including atherosclerotic plaque rupture and vasculitis.

The first six proformas each comprise of a single table with five columns on one page. This can be printed or accessed electronically, completed by the appropriate oncology or haemato-oncology healthcare professional with the patient, and filed in the patient’s medical records (paper or digital):

- Column 1 lists the CV risk factors
- Column 2 is the box to complete if present (yes/no)
- Column 3 is the weighting of the risk factor (medium, high or very high)
- Column 4 has the level of evidence (LoE) supporting the inclusion and weighting based on the standard LoE definitions used in professional cardiology and oncology guidelines; and
- Column 5 has references for the publications providing evidence for the risk factor having predictive value pre-treatment for future CV adverse events which supports inclusion and level of risk weighting.

The conceptual background of these recommendations is that both patient-related as well as therapy-related factors contribute to the CV risk.^1,85^ The clinical and demographic variables contributing to increased CV risk can be divided into risk factor classes which are similar for the six cancer-related treatments associated with CVD and HF in particular. The CV risk factor classes included in the first six proformas are:

- Pre-existing CVD
- Elevated circulating cardiac biomarkers pre-treatment (if measured)
- Demographic and co-existing medical conditions recognised as CV risk factors
- Previous cardiotoxic cancer treatment
- Lifestyle-related CV risk factors.

Each risk factor class includes a range of risk factors or variables identified as contributing to CV risk for patients receiving the specific cancer therapy according to the evidence available and expert opinion.

Once completed, a risk level can be calculated from the summary using the following simple rules:

1. Patients with no risk factors are ‘low risk’
2. Patients with one or more risk factors are categorised according to the highest risk factor present:
  - Patients with one or more very high risk factors—their risk level is ‘very high’
  - Patients with one or more high risk factors—their risk level is ‘high’
3. Medium risk factors are given a point weighting as medium^1^ or medium^2^
  - Patients with one medium^1^ risk factor only are ‘low risk’
  - Patients with a single medium^2^ risk factor or more than one medium^1^ risk factor with points totalling 2–4 are ‘medium risk’
  - Patients with several medium risk factors with points totalling 5 or more points are ‘high risk’

Evidence for defining the absolute risk is limited or absent for each risk factor for every drug class. Based on discussion and expert opinion, the risk of future cardiotoxicity for each of the risk groups can be considered as follows: low risk <2%, medium risk 2–9%, high risk 10–19%, very high risk ≥20%. These should be considered a guide and future studies are needed to validate and refine these ranges and risk weighting.

The seventh baseline CV risk assessment proforma relates to ADT including GnRH agonists and other anti-androgens (e.g. 17*α*-hydroxylase inhibitors) used for prostate cancer. The risks relate to the development of atherosclerotic vascular disease, and there are several established CV risk calculators for MI and stroke associated with atherosclerosis (Table 9 and ADT baseline CV risk assessment proformas).^86–88^ The risk categories are different from those for the other oncology drugs as they are based on the 10-year risk of events. Several studies have shown that, particularly for prostate cancer patients who have a mean age >60 years and frequently have concomitant coronary artery disease, that GnRH agonists given as ADT increase CV risk and mortality, and preventative strategies are needed.^89^ Whilst these CV risk calculators were not specifically developed for cancer patients receiving GnRH agonists or other ADT, and frequently excluded patients with active cancer, they are established from large population studies and included in the ESC guidelines for CVD prevention and are also included in many national cardiology society guidelines. The risk calculators collect various parameters associated with future risk of atherosclerosis-related CVD, although the specific parameters required vary between the different risk calculators (online supplementary Table S1). It was the consensus of the authors to recommend the use of these established CV risk calculators specifically for patients receiving ADT including GnRH agonists for prostate cancer which have an increased risk of MI and stroke. The coronary heart disease risk level can then be calculated using the online web-based calculator for the risk score as follows:

- <10% 10-year risk = low risk level
- 10–19% 10-year risk = medium risk level
- ≥20% 10-year risk = high risk level

The result should be communicated to the patient and to the appropriate healthcare professionals (primary care physician, cardiologist, cardio-oncologist) to address modifiable CV risk factors according to ESC guidelines for CVD prevention.^86^ These are primary prevention CV risk calculators and are only suitable for cancer patients scheduled to receive ADT who have not previously presented with the clinical manifestations of atherosclerotic disease. Any prostate cancer patient with a previous history of CVD is high risk and should be evaluated by an appropriate healthcare professional to review their symptom status and CV risk factor control. These CV risk calculators are not suitable for other cardiotoxic cancer therapies where there is an increased risk of HF, hypertension, QT prolongation and other CVDs. In addition, data on the increased CV risk in women receiving GnRH agonists for breast or ovarian cancer are lacking and therefore this proforma is currently only applicable to men with prostate cancer scheduled to receive a GnRH agonist.

We recommend completion of the baseline CV risk assessment proformas in all patients scheduled to receive one of the seven oncology drug classes with potential cardiotoxicity listed in Table 1. This can be performed after the decision has been made by the treating oncologist or haematologist to start a potentially cardiotoxic cancer treatment. It is important to emphasise that this needs to be completed promptly so that cancer treatment is not delayed and can be commenced safely. In emergency scenarios, guideline-based cancer treatment should be commenced and the baseline CV risk assessment proformas can be completed once clinical stability has been achieved (e.g. CML presenting with blast crisis, solid tumours presenting with acute oncological emergencies).

Following completion of the baseline CV risk assessment proformas the risk level should be recorded in the patient’s medical records, reviewed by the treating oncologist or haemato-oncologist and communicated to the patient and their primary care physician. The specific treatment pathways for each of the drug categories and risk levels is beyond the scope of this position statement and will be addressed in a future HFA position statement, but the authors recommend, conceptually, the following general principles until more detailed guidance is available:

- *Low risk* level cancer patients continue with treatment with CV surveillance as appropriate according to local, national and international guidelines.
- *Medium risk* cancer patients require closer monitoring of CV health during treatment or consideration for referral for a cardio-oncology or cardiology assessment.
- *High* and *very high risk* level patients are referred for a cardio-oncology or cardiology assessment, ideally in a specialist cardio-oncology service (if available) to optimise management of their pre-existing CVD and modifiable CV risk factors, and provide a personalised management plan for surveillance during cancer treatment.

It is important that pathways exist to minimise the time delay from risk assessment and referral to cardiology clinical assessment, and the decision and management plan are communicated to the referring oncology or haemato-oncology team promptly to prevent any delay in starting cancer treatment, following the core principles of a cardio-oncology service.^2^ The timing and nature of CV surveillance recommendations will depend upon various factors including the cardiotoxicity profile of the cancer therapy required (Table 1), the risk factors contributing to the risk level calculation and patient preference. CV imaging and cardiac biomarkers are available for surveillance and detection of early cardiotoxicity, and their role in cancer patients receiving potentially cardiotoxic cancer therapies and surveillance algorithms are the topic of two HFA position statements (in preparation).

We recommend that following implementation of these risk proformas, which could be digital or paper-based depending upon local medical records, an audit and review of the risk stratification process is performed to identify the frequency of application, percentage of risk assessments completed, the actions taken from the assessment and how that conforms to local pathways and standards of care. Oncologists and haemato-oncologists should identify cardiologists with whom to collaborate in setting up pathways of care for their high risk and very high risk patients. If cardiology support is not available locally, then whilst identifying regional or national options these risk proformas should provide a guide for oncologists to consider alternative, non-cardiotoxic cancer therapies in patients identified as high risk or very high risk where alternative treatment options are available. In the long term collection of outcome data, and comparison to retrospective datasets regarding CV events, could be considered. We suggest collaborative studies between centres implementing these risk stratification proformas to assess their impact in reducing CV complications of cancer therapies as well as changes in the overall cancer and CV outcomes. Large datasets can also serve to refine the weighting of risk for the different parameters for each cancer drug class, with the ultimate aim to improve the sensitivity and predictive value.

## Conclusions and future directions

Cardiology and oncology professional society guidelines and expert position statements on CVD in cancer patients uniformly recommend baseline CV risk assessment for oncology patients scheduled to receive potentially cardiotoxic cancer therapies.^1,4–6^ Here we present proformas for baseline CV risk assessment which can be employed by oncology and haemato-oncology services for patients scheduled to receive one of seven cardiotoxic cancer therapies. Assessment of baseline CV risk is part of a personalised approach to care for cancer patients. The identification of cancer patients who are at an increased risk of CV complications in a timely manner is important so that appropriate measures can be implemented to eliminate or at least mitigate their CV risk and ensure, where possible, that cancer patients receive their treatment safely. There is the potential for these proformas to be electronic with semi-automated population of the fields from the electronic patient record if a suitable platform exists. Future studies are required to validate and refine these proformas, including the specific weighting of each risk factor and the addition of new risk factors as they are identified. The impact of proformas upon overall survival and both CV-related and cancer-related outcomes and mortality needs to be assessed as well. The long-term goal is to improve both oncology and CV outcomes for this patient population through a personalised approach to CV risk, which should allow cancer patients to complete their evidence-based cancer treatments free from CV toxicity and CVD, leading to an improvement in overall survival.

## Supplementary Material

Suppl Table 7

Suppl Table 6

Suppl Table 2

Suppl Table 4

Suppl Table 5

Suppl Table 3

Suppl Table 1

## Figures and Tables

**Table 1:** Cancer therapy classes identified for cardiovascular baseline risk assessment and associated cardiovascular toxicity

Cancer treatment class	Cancer indication	Treatment-related CV toxicity

**Anthracycline chemotherapy**	Breast cancer, lymphoma, acute leukaemia, sarcoma	Heart failure
(doxorubicin, epirubicin, daunorubicin, idarubicin)		Asymptomatic LVSD
		Atrial and ventricular arrhythmias
**HER2-targeted therapies**	HER2+ breast cancer	Heart failure
(trastuzumab, pertuzumab, trastuzumab emtansine (T-DM1), lapatinib, neratinib, tucatinib)	HER2+ gastric cancer	Asymptomatic LVSD
		Hypertension
**VEGF inhibitors**	VEGF TKIs: renal cancer, hepatocellular cancer, thyroid cancer, colon cancer, sarcoma, GIST	Hypertension
TKIs (sunitinib, pazopanib, sorafenib, axitinib, tivozanib, cabozantinib, regorafenib, lenvatinib, vandetinib) and antibodies (bevacizumab, ramucirumab)		Heart failure
		Asymptomatic LVSD
	Antibodies: breast cancer, ovarian cancer, gastric cancer, gastro-oesophageal cancer, colon cancer	Myocardial ischaemia and infarction
		QTc prolongation
**Multi-targeted kinase inhibitors:**	Chronic myeloid leukaemia	Arterial thrombosis
**second and third generation BCR-ABL TKIs**		(myocardial infarction, stroke and PAOD^a^)
		Venous thromboembolism
(ponatinib, nilotinib, dasatinib, bosutinib)		Hypertension
		Heart failure and asymptomatic LVSD
		Atherosclerosis^b^
		QTc prolongation^b^
		Pulmonary hypertension^c^
**Proteasome inhibitors**	Multiple myeloma	Heart failure^d^
(carfilzomib, bortezomib, ixazomib)		Asymptomatic LVSD^d^
**Immunomodulatory drugs**		Myocardial ischaemia and infarction
(lenalidomide, pomalidomide)		Atrial and ventricular arrhythmias
		Venous thromboembolism
		Arterial thrombosis
		Hypertension
**Combination RAF and MEK inhibitors**	Raf mutant melanoma	Heart failure and asymptomatic LVSD
(dabrafenib + trametinib, vemurafenib + cobimetinib, encorafenib + binimetinib)		Hypertension
		QTc prolongation^e^
**Androgen deprivation therapies**	Prostate cancer	Atherosclerosis
**GnRH agonists** (goserelin, leuprorelin)	ER+ breast cancer^f^	Myocardial ischaemia and infarction
**Antiandrogrens** (abiraterone)		Diabetes mellitus
		Hypertension
**Immune checkpoint inhibitors:**	Melanoma (metastatic and adjuvant)	Myocarditis including fulminant myocarditis
**anti-programmed cell death 1 inhibitors** (nivolumab, pembrolizumab)	Metastatic renal cancer, non-small cell lung cancer, small cell lung cancer, refractory Hodgkin’s lymphoma, metastatic triple negative breast cancer, metastatic urothelial cancer, liver cancer, MMR-deficient cancer	Pericarditis
		Non-inflammatory heart failure
**anti-cytotoxic T-lymphocyte-associated protein 4 inhibitor** (ipilimumab)		Ventricular arrhythmias
		AV block
**anti-programmed death-ligand 1 inhibitors** (avelumab, atezolizumab, durvalumab)		Acute coronary syndromes including atherosclerotic plaque rupture and vasculitis

AV, atrio-ventricular; CV, cardiovascular; ER, oestrogen receptor; GIST, gastro-intestinal stromal tumour; GnRH, gonadotropin release hormone; LVSD, left ventricular systolic dysfunction; MMR, mismatch repair; PAOD, peripheral arterial occlusive disease; TKI, tyrosine kinase inhibitor; VEGF, vascular endothelial growth factor.

a Associated with ponatinib.

b Associated with ponatinib and nilotinib.

c Associated with dasatinib.

d Associated with carfilzomib.

e Associated with vemurafenib and cobimetinib.

f The risk scores for androgen deprivation therapies in this position statement relate to androgen deprivation therapies for prostate cancer only.

**Table 2:** Baseline cardiovascular risk stratification proforma for anthracycline chemotherapy

Risk factor	Score	Level of evidence	References

Previous cardiovascular disease			
Heart failure or cardiomyopathy	Very high	B	10,11
Severe valvular heart disease	High	C	11
Myocardial infarction or previous coronary revascularisation (PCI or CABG)	High	C	10–12
Stable angina	High	C	10–12
Baseline LVEF <50%	High	B	10
Borderline LVEF 50–54%	Medium^2^	C	
Cardiac biomarkers (where available)			
Elevated baseline troponin^a^	Medium^1^	C	13–15
Elevated baseline BNP or NT-proBNP^a^	Medium^1^	C	16,17
Demographic and cardiovascular risk factors			
Age ≥80 years	High	B	10,12,18
Age 65–79 years	Medium^2^	B	10,18–20
Hypertension^b^	Medium^1^	B	11,12,21
Diabetes mellitus^c^	Medium^1^	C	10–12
Chronic kidney disease^d^	Medium^1^	C	
Previous cardiotoxic cancer treatment			
Previous anthracycline exposure	High	B	18–20,22–25
Prior radiotherapy to left chest or mediastinum	High	C	20,22,23,26,27
Previous non-anthracycline-based chemotherapy	Medium^1^	C	24,25,28
Lifestyle risk factors			
Current smoker or significant smoking history	Medium^1^	C	23
Obesity (BMI >30 kg/m^2^)	Medium^1^	C	20,29,30
**Risk level**			

BMI, body mass index; BNP, brain natriuretic peptide; CABG, coronary artery bypass graft; LVEF, left ventricular ejection fraction; NT-proBNP, N-terminal pro-brain natriuretic peptide; PCI, percutaneous coronary intervention.

Low risk = no risk factor OR one medium^1^ risk factor; Medium risk = medium risk factors with a total of 2–4 points; High risk = medium risk factors with a total of ≥5 points OR any high risk factor; Very high risk = any very high risk factor.

a Elevated above the upper limit of normal for local laboratory reference range.

b Systolic blood pressure >140 mmHg or diastolic blood pressure >90 mmHg, or on treatment.

c Glycated haemoglobin >7.0% or >53 mmol/mol, or on treatment.

d Estimated glomerular filtration rate <60 mL/min/1.73 m^2^.

Please see online supplementary Table S2 for the 1 page printable version for clinical use.

**Table 3:** Baseline cardiovascular risk stratification proforma for HER2-targeted cancer therapies (trastuzumab, pertuzumab, T-DM1, lapatinib, neratinib)

Risk factor	Score	Level of evidence	References

Previous cardiovascular disease			
Heart failure or cardiomyopathy	Very high	C	31
Myocardial infarction or CABG	High	B	31,32
Stable angina	High	B	31–34
Severe valvular heart disease	High	C	31
Baseline LVEF <50%	High	C	
Borderline LVEF 50–54%	Medium^2^	B	35–37
Arrhythmia^a^	Medium^2^	C	31,32
Cardiac biomarkers (where available)			
Elevated baseline troponin^b^	Medium^2^	B	38,39
Elevated baseline BNP or NT-proBNP^b^	Medium^2^	C	17
Demographic and cardiovascular risk factors			
Age ≥80 years	High	B	32,33
Age 65–79 years	Medium^2^	B	35,36,40,41
Hypertension^c^	Medium^1^	B	32–36,42,43
Diabetes mellitus^d^	Medium^1^	C	31,32,42
Chronic kidney disease^e^	Medium^1^	C	32
Current cancer treatment regimen			
Includes anthracycline before HER2-targeted therapy	Medium^1f^	B	32,40,41,43–45
Previous cardiotoxic cancer treatment			
Prior trastuzumab cardiotoxicity	Very high	C	
Prior (remote) anthracycline exposure^g^	Medium^2^	B	42
Prior radiotherapy to left chest or mediastinum	Medium^2^	C	41,46,47
Lifestyle risk factors			
Current smoker or significant smoking history	Medium^1^	C	34
Obesity (BMI >30 kg/m^2^)	Medium^1^	C	29,34,43,45
**Risk level**			

BMI, body mass index; BNP, brain natriuretic peptide; CABG, coronary artery bypass graft; LVEF, left ventricular ejection fraction; NT-proBNP, N-terminal pro-brain natriuretic peptide.

Low risk = no risk factor OR one medium^1^ risk factor; Medium risk = medium risk factors with a total of 2–4 points; High risk = medium risk factors with a total of ≥5 points OR any high risk factor; Very high risk = any very high risk factor.

a Atrial fibrillation, atrial flutter, ventricular tachycardia, or ventricular fibrillation.

b Elevated above the upper limit of normal for local laboratory reference range.

c Systolic blood pressure >140 mmHg or diastolic blood pressure >90 mmHg, or on treatment.

d Glycated haemoglobin >7.0% or >53 mmol/mol, or on treatment.

e Estimated glomerular filtration rate <60 mL/min/1.73 m^2^.

f High risk if anthracycline chemotherapy and trastuzumab delivered concurrently.

g Previous malignancy (not current treatment protocol).

Please see online supplementary Table S3 for the 1 page printable version for clinical use.

**Table 4:** Baseline cardiovascular risk stratification proforma for vascular endothelial growth factor inhibitors

Risk factor	Score	Level of evidence	References

Previous cardiovascular disease			
Heart failure or cardiomyopathy	Very high	C	48–50
Arterial vascular disease (IHD, PCI, CABG, stable angina, TIA, stroke, PVD)	Very high	C	50–52
Venous thrombosis (DVT or PE)	High	C	
Baseline LVEF <50%	High	C	
Borderline LVEF 50–54%	Medium^2^	C	
QTc ≥480 ms	High	C	
450 ms ≤ QTc <480 ms (men) 460 ms ≤ QTc <480 ms (women)	Medium^2^	C	
Arrhythmia^a^	Medium^2^	C	50
Cardiac biomarkers (where available)			
Elevated baseline troponin^b^	Medium^1^	C	50
Elevated baseline BNP or NT-proBNP^b^	Medium^1^	C	53
Demographic and cardiovascular risk factors			
Age ≥75 years	High	C	54–56
Age 65–74 years	Medium^1^	C	48,54,56
Hypertension^c^	High	C	48,50–52,54,55
Diabetes mellitus^d^	Medium^1^	C	50
Hyperlipidaemia^e^	Medium^1^	C	49,50
Chronic kidney disease^f^	Medium^1^	C	57
Proteinuria	Medium^1^	C	
Previous cardiotoxic cancer treatment			
Prior anthracycline exposure	High	C	
Prior radiotherapy to left chest or mediastinum	Medium^1^	C	
Lifestyle risk factors			
Current smoker or significant smoking history	Medium^1^	C	50
Obesity (BMI >30 kg/m^2^)	Medium^1^	C	50,54,58
**Risk level**			

BMI, body mass index; BNP, brain natriuretic peptide; CABG, coronary artery bypass graft; DVT, deep vein thrombosis; IHD, ischaemic heart disease; LVEF, left ventricular ejection fraction; NT-proBNP, N-terminal pro-brain natriuretic peptide; PCI, percutaneous coronary intervention; PE, pulmonary embolism; PVD, peripheral vascular disease; TIA, transient ischaemic attack.

Low risk = no risk factor OR one medium^1^ risk factor; Medium risk = medium risk factors with a total of 2–4 points; High risk = medium risk factors with a total of ≥5 points OR any high risk factor; Very high risk = any very high risk factor.

a Atrial fibrillation, atrial flutter, ventricular tachycardia, or ventricular fibrillation.

b Elevated above the upper limit of normal for local laboratory reference range.

c Systolic blood pressure >140 mmHg or diastolic blood pressure >90 mmHg, or on treatment.

d Glycated haemoglobin >7.0% or >53 mmol/mol, or on treatment.

e Non-high-density lipoprotein cholesterol level >3.8 mmol/L (>145 mg/dL).

f Estimated glomerular filtration rate <60 mL/min/1.73 m^2^.

Please see online supplementary Table S4 for the 1 page printable version for clinical use.

**Table 5:** Baseline cardiovascular risk stratification proforma for multi-targeted kinase inhibitors for chronic myeloid leukaemia including second and third generation BCR-ABL tyrosine kinase inhibitors

Risk factor	Score	Level of evidence	References

Previous cardiovascular disease			
Arterial vascular disease (IHD, PCI, CABG, stable angina, TIA, stroke, PVD)	Very high	C	59,60
Arterial thrombosis with TKI	Very high	C	
Heart failure or LVSD	High	C	
BCR-ABL TKI-mediated LVSD	High	C	
Abnormal ABPI^f^	High	C	
Pulmonary arterial hypertension^g^	High	C	
Baseline LVEF <50%	High	C	
Venous thromboembolism (DVT/PE)	Medium^2^	C	60,61
Arrhythmia^a^	Medium^2^	C	
QTc ≥ 480 ms	High	C	
450 ms ≤ QTc < 480 ms (men) 460 ms ≤ QTc < 480 ms (women)	Medium^2^	C	
Demographic and other cardiovascular risk factors			
Cardiovascular disease 10-year risk score >20%	High	B	62
Hypertension^b^	Medium^2^	B	59–61
Diabetes^c^	Medium^1^	B	63
Hyperlipidaemia^d^	Medium^1^	B	60,61
Age ≥75 years	High	C	
Age 65–74 years	Medium^2^	B	61
Age ≥60 years	Medium^1^	B	61
Chronic kidney disease^e^	Medium^1^	C	
Family history of thrombophilia	Medium^1^	C	
Lifestyle and other factors			
Current smoker or significant smoking history	High	B	60
Obesity (BMI >30kg/m^2^)	Medium^1^	C	
**Risk level**			

ABPI, ankle–brachial pressure index; BMI, body mass index; CABG, coronary artery bypass graft; DVT, deep vein thrombosis; IHD, ischaemic heart disease; LVEF, left ventricular ejection fraction; LVSD, left ventricular systolic dysfunction; PCI, percutaneous coronary intervention; PCI, percutaneous coronary intervention; PE, pulmonary embolism; PVD, peripheral vascular disease; TIA, transient ischaemic attack; TKI, tyrosine kinase inhibitor.

Low risk = no risk factor OR one medium^1^ risk factor; Medium risk = medium risk factors with a total of 2–4 points; High risk = medium risk factors with a total of ≥5 points OR any high risk factor; Very high risk = any very high risk factor.

a Atrial fibrillation, atrial flutter, ventricular tachycardia, or ventricular fibrillation.

b Systolic blood pressure >140 mmHg or diastolic blood pressure >90 mmHg, or on treatment.

c Glycated haemoglobin >7.0% or >53 mmol/mol, or on treatment.

d Non-high-density lipoprotein cholesterol level >3.8mmol/L (>145mg/dL).

e Estimated glomerular filtration rate <60mL/min/1.73 m^2^.

f ABPI ≤0.9.

g Peak systolic pulmonary artery pressure at rest ≥35 mmHg when estimated non-invasively on echocardiography.

Please see online supplementary Table S5 for the 1 page printable version for clinical use.

**Table 6:** Baseline cardiovascular risk stratification proforma for proteasome inhibitors and immunomodulatory agents for multiple myeloma

Risk factor	Score	Level of evidence	References

Previous cardiovascular disease			
Heart failure or cardiomyopathy	Very high	C	64
Prior proteasome inhibitor cardiotoxicity	Very high	C	
Venous thrombosis (DVT or PE)	Very high	C	64
Cardiac amyloidosis	Very high	C	
Arterial vascular disease (IHD, PCI, CABG, stable angina, TIA, stroke, PVD)	Very high	C	64
Prior immunomodulatory drug CV toxicity	High	B	65
Baseline LVEF <50%	High	C	
Borderline LVEF 50–54%	Medium^2^	C	
Arrhythmia^a^	Medium^2^	C	64
Left ventricular hypertrophy^b^	Medium^1^	C	
Cardiac biomarkers (where available)			
Elevated baseline troponin^c^	Medium^2^	C	
Elevated baseline BNP or NT-proBNP^c^	High	B	66
Demographic and cardiovascular risk factors			
Age ≥75 years	High	C	
Age 65–74 years	Medium^1^	C	
Hypertension^d^	Medium^1^	C	64,67
Diabetes mellitus^e^	Medium^1^	C	
Hyperlipidaemia^f^	Medium^1^	C	64
Chronic kidney disease^g^	Medium^1^	C	
Family history of thrombophilia	Medium^1^	C	
Previous cardiotoxic cancer treatment			
Prior anthracycline exposure	High	C	68
Prior thoracic spine radiotherapy	Medium^1^	C	68
Current myeloma treatment			
High-dose dexamethasone >160 mg/month	Medium^1^	C	
Lifestyle risk factors			
Current smoker or significant smoking history	Medium^1^	C	67
Obesity (BMI >30 kg/m^2^)	Medium^1^	C	
**Risk level**			

BMI, body mass index; BNP, brain natriuretic peptide; CABG, coronary artery bypass graft; DVT, deep vein thrombosis; IHD, Ischaemic heart disease; LVEF, left ventricular ejection fraction; NT-proBNP, N-terminal pro-brain natriuretic peptide; PCI, percutaneous coronary intervention; PE, pulmonary embolism; PVD, peripheral vascular disease; TIA, transient ischaemic attack.

Low risk = no risk factor OR one medium^1^ risk factor; Medium risk = medium risk factors with a total of 2–4 points; High risk = medium risk factors with a total of ≥5 points OR any high risk factor; Very high risk = any very high risk factor.

a Atrial fibrillation, atrial flutter, ventricular tachycardia, or ventricular fibrillation.

b Left ventricular wall thickness >1.2 cm.

c Elevated above the upper limit of normal for local laboratory reference range.

d Systolic blood pressure >140 mmHg or diastolic blood pressure >90 mmHg, or on treatment.

e Glycated haemoglobin >7.0% or >53 mmol/mol or on treatment.

f Non-high-density liporotein cholesterol level >3.8mmol/L (>145mg/dL).

g Estimated glomerular filtration rate <60mL/min/1.73 m^2^.

Please see online supplementary Table S6 for the 1 page printable version for clinical use.

**Table 7:** Baseline cardiovascular risk stratification proforma for combination RAF and MEK inhibitors (dabrafenib + trametinib, vemurafenib + cobimetinib, encorafenib + binimetinib)

Risk factor	Score	Level of evidence

Previous cardiovascular disease		
Heart failure or cardiomyopathy	Very high	C
Myocardial infarction or CABG	High	C
Stable angina	High	C
Severe valvular heart disease	High	C
Borderline LVEF 50–54%	Medium^2^	C
Arrhythmia^a^	Medium^1^	C
Cardiac biomarkers (where available)		
Elevated baseline troponin^b^	Medium^2^	C
Elevated baseline BNP or NT-proBNP^b^	Medium^2^	C
Demographic and cardiovascular risk factors		
Age ≥65 years	Medium^1^	C
Hypertension^c^	Medium^2^	C
Diabetes mellitus^d^	Medium^1^	C
Chronic kidney disease^e^	Medium^1^	C
Previous cardiotoxic cancer treatment		
Prior anthracycline exposure^f^	High	C
Prior radiotherapy to left chest or mediastinum	Medium^2^	C
Lifestyle risk factors		
Current smoker or significant smoking history	Medium^1^	C
Obesity (BMI >30 kg/m^2^)	Medium^1^	C
**Risk level**		

BMI, body mass index; BNP, brain natriuretic peptide; CABG, coronary artery bypass graft; LVEF, left ventricular ejection fraction; NT-proBNP, N-terminal pro-brain natriuretic peptide.

Low risk = no risk factor OR one medium^1^ risk factor; Medium risk = medium risk factors with a total of 2–4 points; High risk = medium risk factors with a total of ≥5 points OR any high risk factor; Very high risk = any very high risk factor.

a Atrial fibrillation, atrial flutter, ventricular tachycardia, or ventricular fibrillation.

b Elevated above the upper limit of normal for local laboratory reference range.

c Systolic blood pressure >140 mmHg or diastolic blood pressure >90 mmHg, or on treatment.

d Glycated haemoglobin >7.0% or >53 mmol/mol, or on treatment.

e Estimated glomerular filtration rate <60 mL/min/1.73 m^2^.

f Previous malignancy.

Please see online supplementary Table S7 for the 1 page printable version for clinical use.

**Table 8:** Baseline cardiovascular risk stratification proforma for androgen deprivation therapies including gonadotrophin-releasing hormone agonists (goserelin, leuprolide) and anti-androgen therapies (abiraterone) for prostate cancer

Clinical risk score^a^	Score

Known pre-existing cardiovascular disease (CVD)^b^ or CVD 10-year risk score ≥20%	High
CVD 10-year risk score ≥10% to <20%	Medium
CVD 10-year risk score <10%	Low

CVD, cardiovascular disease.

Risk factors and variables required: age, gender, ethnic group, height, weight, social class indicator (Townsend quintile), smoking status (current, ex- or non-smoker), total cholesterol, high-density lipoprotein cholesterol, systolic blood pressure (mmHg), diabetes status (yes/no), family history of premature CVD (before 60 years) (yes/no), chronic kidney disease (yes/no), atrial fibrillation (yes/no), systemic inflammatory disease (e.g. rheumatoid arthritis, psoriasis) (yes/no).

a For validated CVD risk scores, see Table 9.

b Prior symptomatic coronary artery disease, carotid artery disease or peripheral artery disease, e.g. stable angina, acute myocardial infarction, transient ischaemic attack/stroke, ischaemic claudication.

**Table 9:** Atherosclerosis-related cardiovascular risk calculators

Risk score	Website

ESC HeartScore	www.heartscore.org
QRISK®3	https://qrisk.org/three
JBS3 risk score (2014)	http://www.jbs3risk.com
ACC/AHA pooled cohort CV risk calculator (2013)	http://www.cvriskcalculator.com

ACC, American College of Cardiology; AHA, American Heart Association; ESC, European Society of Cardiology; JBS, Joint British Societies.
