# Supplementary material for: Baseline cardiovascular risk assessment in cancer patients scheduled to receive cardiotoxic cancer therapies: a position statement and new risk assessment tools from the Cardio-Oncology Study Group of the Heart Failure Association of the European Society of Cardiology in collaboration with the International Cardio-Oncology Society
Source: Eur J Heart Fail. Author manuscript; Available in PMC 2021 Apr 3. (PMC8019326; doi:10.1002/ejhf.1920)
Supplement: Suppl Table 2 [file NIHMS1663326-supplement-Suppl_Table_2.pdf]

HFA European Society of Cardiology

ESC Council Cardio-Oncology

International Cardio Oncology Society 2009

BASELINE CARDIO-ONCOLOGY RISK ASSESSMENT

ANTHRACYCLINE CHEMOTHERAPY

Risk Factor	Risk Factor Present	Score	Level of Evidence
**Previous cardiovascular disease**			
Heart failure or cardiomyopathy		**VERY HIGH**	**B**
Severe valvular heart disease		**HIGH**	**C**
Myocardial infarction or previous coronary revascularisation (PCI or CABG)		**HIGH**	**C**
Stable angina		**HIGH**	**C**
Baseline LVEF <50%		**HIGH**	**B**
Borderline LVEF 50-54%		**MEDIUM[2]**	**C**
**Cardiac Biomarkers (where available)**			
Elevated baseline troponin*		**MEDIUM[1]**	**C**
Elevated baseline BNP or NT-proBNP*		**MEDIUM[1]**	**C**
**Demographic and cardiovascular risk factors**			
Age ≥80 years		**HIGH**	**B**
Age 65-79 years		**MEDIUM[2]**	**B**
Hypertension ✎		**MEDIUM[1]**	**B**
Diabetes mellitus ✦		**MEDIUM[1]**	**C**
Chronic kidney disease ⅄		**MEDIUM[1]**	**C**
**Previous cardiotoxic cancer treatment**			
Previous anthracycline exposure		**HIGH**	**B**
Prior radiotherapy to left chest or mediastinum		**HIGH**	**C**
Previous non-anthracycline-based chemotherapy		**MEDIUM[1]**	**C**
**Lifestyle risk factors**			
Current smoker or significant smoking history		**MEDIUM[1]**	**C**
Obesity (BMI>30)		**MEDIUM[1]**	**C**
**RISK LEVEL**			

LEGEND

BMI = Body mass index
BNP = Brain natriuretic peptide
CABG = Coronary artery bypass graft
LVEF = Left ventricular ejection fraction
NT-proBNP = N-terminal pro-brain natriuretic peptide
* Elevated above the upper limit of normal for local laboratory reference range
✎ Systolic blood pressure (BP) >140mmg Hg or diastolic BP >90mm Hg, or on treatment
✦ HbA1c >7.0% or >53mmol/mol or on treatment
⅄ Estimated glomerular filtration rate <60ml/min/1.73m^2

LOW RISK = no risk factor **OR** one MEDIUM1 RF
MEDIUM RISK = MEDIUM RFs with a total of 2-4 points
HIGH RISK = MEDIUM RFs with a total of ≥5 points **OR** any HIGH RF
VERY HIGH RISK = any VERY HIGH RF
