# Supplementary material for: Baseline cardiovascular risk assessment in cancer patients scheduled to receive cardiotoxic cancer therapies: a position statement and new risk assessment tools from the Cardio-Oncology Study Group of the Heart Failure Association of the European Society of Cardiology in collaboration with the International Cardio-Oncology Society
Source: Eur J Heart Fail. Author manuscript; Available in PMC 2021 Apr 3. (PMC8019326; doi:10.1002/ejhf.1920)
Supplement: Suppl Table 4 [file NIHMS1663326-supplement-Suppl_Table_4.pdf]

HFA European Society of Cardiology | ESC Council Cardio-Oncology | International Cardio Oncology Society 2009

BASELINE CARDIO-ONCOLOGY RISK ASSESSMENT

VEGF inhibitors

Risk Factor	Risk Factor Present	Score	Level of Evidence
**Previous cardiovascular disease**			
Heart failure or cardiomyopathy		**VERY HIGH**	**C**
Arterial vascular disease (IHD, PCI, CABG, stable angina, TIA, stroke, PVD)		**VERY HIGH**	**C**
Venous thrombosis (DVT or PE)		**HIGH**	**C**
Baseline LVEF <50%		**HIGH**	**C**
Borderline LVEF 50-54%		**MEDIUM[2]**	**C**
QTc ≥ 480ms		**HIGH**	**C**
450ms ≤ QTc < 480ms (men) 460ms ≤ QTc < 480ms (women)		**MEDIUM[2]**	**C**
Arrhythmia✧		**MEDIUM[2]**	**C**
**Cardiac Biomarkers (where available)**			
Elevated baseline troponin*		**MEDIUM[1]**	**C**
Elevated baseline BNP or NT-proBNP*		**MEDIUM[1]**	**C**
**Demographic and cardiovascular risk factors**			
Age ≥75 years		**HIGH**	**C**
Age 65-74 years		**MEDIUM[1]**	**C**
Hypertension ℘		**HIGH**	**C**
Diabetes mellitus✦		**MEDIUM[1]**	**C**
Hyperlipidaemia Ø		**MEDIUM[1]**	**C**
Chronic kidney disease ⅄		**MEDIUM[1]**	**C**
Proteinuria		**MEDIUM[1]**	**C**
**Previous cardiotoxic cancer treatment**			
Prior anthracycline exposure		**HIGH**	**C**
Prior radiotherapy to left chest or mediastinum		**MEDIUM[1]**	**C**
**Lifestyle risk factors**			
Current smoker or significant smoking history		**MEDIUM[1]**	**C**
Obesity (BMI>30)		**MEDIUM[1]**	**C**
**RISK LEVEL**			

LEGEND
BMI = Body mass index
BNP = Brain natriuretic peptide
CABG = Coronary artery bypass graft
DVT = Deep vein thrombosis
IHD = Ischaemic heart disease
LVEF = Left ventricular ejection fraction
NT-proBNP = N-terminal pro-brain natriuretic peptide
PCI = Percutaneous coronary intervention
PE = Pulmonary embolism
PVD = Peripheral vascular disease

QTc = Corrected QT interval
TIA = Transient ischaemic attack
✧ Atrial fibrillation, atrial flutter, ventricular tachycardia or ventricular fibrillation
* Elevated above the upper limit of normal for local laboratory reference range
℘ Systolic blood pressure (BP) >140mmg Hg or diastolic BP >90mm Hg, or on treatment
✦ HbA1c >7.0% or >53mmol/mol or on treatment
Ø Non-HDL cholesterol level >3.8mmol/L (>145mg/dL)
⅄ Estimated glomerular filtration rate <60ml/min/1.73m^2

LOW RISK = no risk factor **OR** one MEDIUM1 RF
MEDIUM RISK = MEDIUM RFs with a total of 2-4 points
HIGH RISK = MEDIUM RFs with a total of ≥5 points **OR** <u>any</u> HIGH RF
VERY HIGH RISK = <u>any</u> VERY HIGH RF
