# Supplementary material for: Baseline cardiovascular risk assessment in cancer patients scheduled to receive cardiotoxic cancer therapies: a position statement and new risk assessment tools from the Cardio-Oncology Study Group of the Heart Failure Association of the European Society of Cardiology in collaboration with the International Cardio-Oncology Society
Source: Eur J Heart Fail. Author manuscript; Available in PMC 2021 Apr 3. (PMC8019326; doi:10.1002/ejhf.1920)
Supplement: Suppl Table 5 [file NIHMS1663326-supplement-Suppl_Table_5.pdf]

BASELINE CARDIO-ONCOLOGY RISK ASSESSMENT

Multitargeted Kinase inhibitors for CML

2nd and 3rd generation BCR-ABL TYROSINE KINASE INHIBITORS

RISK factor	Risk Factor Present	Score	Level of evidence
**Previous cardiovascular disease**			
Arterial vascular disease (IHD, PCI, CABG, stable angina, TIA, stroke, PVD)		**VERY HIGH**	**C**
Arterial thrombosis with TKI		**VERY HIGH**	**C**
Heart failure or LVSD		**HIGH**	**C**
BCR-ABL TKI-mediated LVSD		**HIGH**	**C**
Abnormal ABPI *		**HIGH**	**C**
Pulmonary Arterial Hypertension **		**HIGH**	**C**
Baseline LVEF <50%		**HIGH**	**C**
Venous thromboembolism (DVT/PE)		**MEDIUM[2]**	**C**
Arrhythmia ✧		**MEDIUM[2]**	**C**
QTc ≥ 480ms		**HIGH**	**C**
450ms ≤ QTc < 480ms (men)<br>460ms ≤ QTc < 480ms (women)		**MEDIUM[2]**	**C**
**Demographic and other CV risk factors**			
CVD 10 year risk score >20%		**HIGH**	**B**
Hypertension ☇		**MEDIUM[2]**	**B**
Diabetes ✦		**MEDIUM[1]**	**B**
Hyperlipidaemia Ø		**MEDIUM[1]**	**B**
Age ≥75 years		**HIGH**	**C**
Age 65-74 years		**MEDIUM[2]**	**B**
Age ≥60 years		**MEDIUM[1]**	**B**
Chronic kidney disease ⅄		**MEDIUM[1]**	**C**
Family History of Thrombophilia		**MEDIUM[1]**	**C**
**Lifestyle and other factors**			
Current smoker or significant smoking history		**HIGH**	**B**
Obesity (BMI>30)		**MEDIUM[1]**	**C**
**RISK LEVEL**			

LEGEND

BMI = Body mass index
CABG = Coronary artery bypass graft
IHD = Ischaemic heart disease
PCI = Percutaneous coronary intervention
PVD = Peripheral vascular disease
TIA = Transient ischaemic attack
LVSD = Left ventricular systolic dysfunction
CVD = Cardiovascular disease
✧ Atrial fibrillation, atrial flutter, ventricular tachycardia or ventricular fibrillation
☇ Systolic blood pressure (BP) >140mmg Hg or diastolic BP >90mm Hg, or on treatment

✦ HbA1c >7.0% or >53mmol/mol or on treatment
Ø Non-HDL cholesterol level >3.8mmol/L (>145mg/dL)
⅄ Estimated glomerular filtration rate <60ml/min/1.73m^2
* ankle-brachial pressure index ≤0.9
** Peak systolic PA pressure at rest ≥35 mmHg when estimated non-invasively via echocardiography

LOW RISK = no risk factor **OR** one MEDIUM1 RF
MEDIUM RISK = MEDIUM RFs with a total of 2-4 points
HIGH RISK = MEDIUM RFs with a total of ≥5 points **OR** <u>any</u> HIGH RF
VERY HIGH RISK = <u>any</u> VERY HIGH RF
