# Supplementary material for: Baseline cardiovascular risk assessment in cancer patients scheduled to receive cardiotoxic cancer therapies: a position statement and new risk assessment tools from the Cardio-Oncology Study Group of the Heart Failure Association of the European Society of Cardiology in collaboration with the International Cardio-Oncology Society
Source: Eur J Heart Fail. Author manuscript; Available in PMC 2021 Apr 3. (PMC8019326; doi:10.1002/ejhf.1920)
Supplement: Suppl Table 3 [file NIHMS1663326-supplement-Suppl_Table_3.pdf]

HFA European Society of Cardiology | ESC Council Cardio-Oncology | International Cardio Oncology Society 2009

BASELINE CARDIO-ONCOLOGY RISK ASSESSMENT

HER2-targeted therapies

TRASTUZUMAB, PERTUZUMAB, T-DM1, LAPATINIB, NERATINIB

Risk Factor	Risk Factor Present	Score	Level of Evidence
**Previous cardiovascular disease**			
Heart failure or cardiomyopathy		**VERY HIGH**	**C**
Myocardial infarction or CABG		**HIGH**	**B**
Stable angina		**HIGH**	**B**
Severe valvular heart disease		**HIGH**	**C**
Baseline LVEF <50%		**HIGH**	**C**
Borderline LVEF 50-54%		**MEDIUM[2]**	**B**
Arrhythmia ✧		**MEDIUM[2]**	**C**
**Cardiac biomarkers (where available)**			
Elevated baseline troponin*		**MEDIUM[2]**	**B**
Elevated baseline BNP or NT-proBNP*		**MEDIUM[2]**	**C**
**Demographic and cardiovascular risk factors**			
Age ≥80 years		**HIGH**	**B**
Age 65-79 years		**MEDIUM[2]**	**B**
Hypertension ⌀		**MEDIUM[1]**	**B**
Diabetes mellitus ✦		**MEDIUM[1]**	**C**
Chronic kidney disease ⅄		**MEDIUM[1]**	**C**
**Current cancer treatment regimen**			
Includes Anthracycline before HER2-targeted therapy		**MEDIUM[1**]**	**B**
**Previous cardiotoxic cancer treatment**			
Prior trastuzumab cardiotoxicity		**VERY HIGH**	**C**
Prior (remote) anthracycline exposure***		**MEDIUM[2]**	**B**
Prior radiotherapy to left chest or mediastinum		**MEDIUM[2]**	**C**
**Lifestyle risk factors**			
Current smoker or significant smoking history		**MEDIUM[1]**	**C**
Obesity (BMI>30)		**MEDIUM[1]**	**C**
**RISK LEVEL**			

LEGEND

BMI = Body mass index
BNP = Brain natriuretic peptide
CABG = Coronary artery bypass graft
LVEF = Left ventricular ejection fraction
NT-proBNP = N-terminal pro-brain natriuretic peptide
✧ Atrial fibrillation, atrial flutter, ventricular tachycardia or ventricular fibrillation
* Elevated above the upper limit of normal for local laboratory reference range
⌀ Systolic blood pressure (BP) >140mmg Hg or diastolic BP >90mm Hg, or on treatment
✦ HbA1c >7.0% or >53mmol/mol or on treatment
⅄ Estimated glomerular filtration rate <60ml/min/$1.73m^2$
** HIGH risk if anthracycline chemotherapy and trastuzumab delivered concurrently
*** Previous malignancy (not current treatment protocol)

LOW RISK = no risk factor **OR** one MEDIUM1 RF
MEDIUM RISK = MEDIUM RFs with a total of 2-4 points
HIGH RISK = MEDIUM RFs with a total of ≥5 points **OR** <u>any</u> HIGH RF
VERY HIGH RISK = <u>any</u> VERY HIGH RF
