# Supplementary material for: Baseline cardiovascular risk assessment in cancer patients scheduled to receive cardiotoxic cancer therapies: a position statement and new risk assessment tools from the Cardio-Oncology Study Group of the Heart Failure Association of the European Society of Cardiology in collaboration with the International Cardio-Oncology Society
Source: Eur J Heart Fail. Author manuscript; Available in PMC 2021 Apr 3. (PMC8019326; doi:10.1002/ejhf.1920)
Supplement: Suppl Table 1 [file NIHMS1663326-supplement-Suppl_Table_1.pdf]

Table S1

CV risk calculators and parameters included in risk calculations for various international risk calculators

**Risk factors and variables required (✓) or optional (✓±):**	**ESC CV SCORE**	**QRISK®3**	**JBS3 risk score**	**ACC/AHA pooled cohort CVD risk calculator**
**Age**	✓	✓	✓	✓
Gender	✓	✓	✓	✓
Systolic BP	✓	✓±	✓	✓
Diastolic BP				✓
Standard deviation of ≥2 most recent systolic BPs		✓±		
Total cholesterol	✓		✓	✓
On blood pressure treatment?		✓	✓	✓
HDL	✓±		✓	✓
Cholesterol/HDL ratio:		✓±		
Smoking status	✓	✓	✓	✓
Ethnicity		✓±	✓±	✓
Diabetes status		✓	✓	✓
Body mass index		✓±	✓	
Angina or heart attack in a 1st degree relative < 60?		✓	✓	
Chronic kidney disease?		✓	✓	
**Atrial fibrillation?**		✓	✓	
Rheumatoid arthritis		✓	✓	
**Do you have migraines?**		✓		
**Systemic lupus erythematosus (SLE)?**		✓		
**Severe mental illness?**		✓		
**On atypical antipsychotic medication?**		✓		
**Regular steroid tablets?**		✓		
**Diagnosis of or treatment for erectile dysfunction**		✓		
UK post code		✓±		
Townsend deprivation index			✓	
**EU country of residence**	✓			
